# Duration of suppression of bone turnover following treatment with zoledronic acid in men with metastatic castration-resistant prostate cancer

**DOI:** 10.4155/fsoa-2017-0094

**Published:** 2017-10-20

**Authors:** Saroj Niraula, Arnoud J Templeton, Francisco Vera-Badillo, Anna Dodd, Zoann Nugent, Anthony M Joshua, Ian F Tannock

**Affiliations:** 1 Department of Medical Oncology and Haematology, CancerCare Manitoba & University of Manitoba, Winnipeg, Canada; 2 Faculty of Medicine, University of Basel, Basel, Switzerland; 3 Department of Medical Oncology and Haematology, Canadian Clinical Trials Group & Queens University, Canada; 4 Department of Medical Oncology and Haematology, St Vincent's Hospital, University of New South Wales, Sydney, Australia; 5 Department of Medical Oncology and Haematology, Princess Margaret Cancer Centre, University of Toronto, Toronto, Canada

**Keywords:** bone metastasis, castration resistant, frequency, prostate cancer, telopeptides, zoledronic acid, zometa

## Abstract

**Aim:**

Zoledronate is approved for use every 3 weeks in men with bone metastases from castrate-resistant prostate cancer (CRPC) but the basis for such frequency is unclear.

**Methods:**

In men with bone metastasis from CRPC we measured the markers of bone turnover - urine and serum telopeptides before the first injection of zoledronate and at four 3-weekly intervals thereafter. Men received further zoledronate treatment after 12 weeks, or earlier if the telopeptides did not meet predefined adequate suppression. The primary end point was the proportion of evaluable subjects with suppressed telopeptides at 12 weeks. Exploratory analyses evaluated predictors of bone turnover suppression and quality-of-life.

**Results:**

31 patients were enrolled. Median age was 70 (range: 53–86) years. 65%, (95% CI: 46–81%) had suppressed telopeptides at 12 weeks. Prior skeletal-related events, chemotherapy, bone surgery and higher baseline levels of telopeptides were associated with shorter duration of telopeptides.

**Conclusion:**

12-weekly zoledronate suppresses bone turnover in the majority of men with bone metastasis from CRPC.

## Lay abstract

Zoledronic acid (ZA) is a bone strengthening drug used for treatment of men with advanced prostate cancer in their bones. Based on initial clinical trials, ZA is approved for use every 3 weeks. However, recent clinical trials suggest similar outcomes when ZA is used every 12 weeks. In this clinical trial, we reaffirm that majority of men with prostate cancer only require every 12 weeks of ZA to achieve a reasonable biochemical response. Less frequent treatments may spare patients of the side effects, inconvenience and cost.

The bisphosphonate zoledronic acid (ZA) has been shown to maintain bone density in men receiving androgen deprivation therapy for prostate cancer and in men without bone metastases. This can be achieved with a 4 mg intravenous dose given annually [1]. In men with castrate-resistant prostate cancer (CRPC) and bone metastases, ZA given every 3 weeks has been demonstrated to delay time to first skeletal-related event (SRE defined as pathologic fracture, spinal cord compression, requirement for radiation therapy or surgery to bone, or change in antineoplastic therapy to treat bone pain) [2,3]. Treatment with ZA has no effect on progression-free or overall survival and is associated with potentially serious side effects including renal impairment, hypocalcemia and osteonecrosis of the jaw.

In the present study, we investigated the duration of suppression of bone turnover following a single dose of ZA in men with bone metastases from CRPC. Our hypothesis was that a single injection of 4 mg ZA would suppress bone turnover in most men for at least 12 weeks.

## Methods

This single arm, prospective cohort study was conducted at the Princess Margaret Cancer Centre, Toronto, Ontario (clinicaltrials.gov identifier NCT01062503). Institutional ethics approval was obtained. Eligible men had CRPC with evidence of bone metastases, were Eastern Cooperative Oncology Group (ECOG) performance status ≤2, had never received bisphosphonates for prostate cancer and had adequate renal and hepatic function. Exclusion criteria included receipt of bisphosphonates within 1 year, infection/abscess on dental exam or dental extraction within 4 weeks prior and other acute problems such as pathological fracture, spinal cord compression or hypocalcemia.

Patients received a single 4 mg intravenous injection of ZA (Zometa^®^, Novartis, Switzerland) in 100 ml saline as a 15-min infusion. Fasting morning blood and urine samples were collected prior to administration of ZA for measurement of the markers of bone turnover uNTX and sCTX (Elecsys, Roche Diagnostics, Basel, Switzerland). Patients had 3-weekly measurements of these markers up to 12 weeks as long as the concentrations of uNTX and sCTX remained suppressed (defined as <50% of baseline value and <100 mmol/mmol creatinine for uNTX and <600 ng/l for sCTX). If the uNTX or sCTX rose above these values, the patient came off study and received further ZA; otherwise they received subsequent ZA at 12 weeks. The primary end point was the proportion of patients with suppression of bone turnover at 12 weeks. Any adverse events were recorded at 3-weekly visits and the relation of such events to treatment with ZA was assessed.

We also measured patients’ quality-of-life (QoL) at baseline, 6 weeks and 12 weeks using the Functional Assessment of Cancer Therapy-Bone Pain (FACT-BP) and the Bone Pain Inventory (BPI) questionnaires [4,5]. Mean FACT-BP and BPI scores are reported descriptively and we sought correlations between changes in uNTX and sCTX concentrations and changes in FACT-BP and BPI scores using Pearson's correlation.

We conducted a univariable proportional hazards regression analysis to determine if baseline variables (age, BMI, prior radiation, prior spinal cord compression, prior chemotherapy, prior SRE, prior surgery to bone and telopeptide concentrations) predicted for suppression of bone turnover at 12 weeks. A multivariable analysis adjusting for covariates was planned. All statistical tests were two sided and statistical significance was defined as p < 0.05. Analyses were carried out using SAS version 9.2 (SAS Institute Inc., NC, USA). No corrections were made for multiple significance testing.

## Results

### Characteristics of participants

From January 2010 to June 2014, 69 men were screened, of whom 29 either declined the study or were ineligible due to use of a bisphosphonate and/or abnormal organ function. Of the remaining 40 men, three withdrew after signing the consent form and six did not have baseline blood work. Baseline characteristics of the 31 eligible men are described in Table 1.

**Table 1.** **Patient demographics and clinical characteristics.**

**Variable**	**n**	**Value**
Age; median (range), years	31	70 (53–86)

Time since diagnosis of bone metastases; median (IQR), months	31	19 (10–40)

BMI; median (IQR), kg/m^2^	31	27.8 (26.2–30.9)

Prior pathological fracture, n (%)	30	4 (13%)

Prior radiotherapy to the bone, n (%)	30	10 (33%)

Prior surgery to bone, n (%)	30	3 (10%)

Prior chemotherapy, n (%)	31	10 (32%)

Baseline uNTX; median (IQR), mmol/mmol creatinine	31	71 (47–189)

Baseline sCTX; median (IQR), ng/l	31	750 (433–1020)

IQR: Interquartile range; uNTX: Urine telopeptide; usCTX: Serum telopeptide.

14 men came off study at or before week 12; nine men had insufficiently suppressed uNTX and sCTX, three were lost to follow-up and two received ZA injections despite suppressed telopeptides. 17 of 26 fully evaluable men therefore had suppression of bone turnover for 12 weeks (65%: 95% CI: 46–81%).

Prior radiotherapy for pain or fracture, prior spinal cord compression, prior chemotherapy, prior SRE and high baseline telopeptide levels were significant predictors of non-suppression of telopeptides at 12 weeks in univariable analysis (Table 2). Multivariable analysis could not be performed due to low event rates.

**Table 2.** **Univariable predictors of failure of telopeptide suppression for 12 weeks following zoledronic acid.**

**Predictor**	**n**	**p-value (χ^2^)**	**Hazard ratio**	**95% CI**	

				**Lower**	**Upper**
Age 70+ vs younger	31	0.82	1.16	0.31	4.32

BMI, per 1 kg/m^2^ higher	31	0.20	0.85	0.66	1.09

Prior radiation for bone pain	30	0.44	1.68	0.45	6.25

Prior radiation for fracture	30	0.003*	7.78	1.99	30.49

Prior spinal cord compression	30	0.02*	2.84	1.14	7.07

Prior chemotherapy	31	0.05*	2.73	1.00	7.43

Prior bone surgery	30	0.13	3.31	0.68	16.04

uNTX >100 mmol/mmol and creatinine and sCTX >600 ng/dl	31	<0.0001*	–^‡^	–	–

Prior SRE	28	0.006*	2.16	1.25	3.72

*Denotes p-values that are statistically significant.

^‡^Denotes the p-value from Cox regression. Hazard ratio could not be calculated and all nine failures occurred in the high-level group.

sCTX: Serum telopeptide; SRE: Skeletal related event; uNTX: Urine telopeptide.

FACT-BP and BPI scores at weeks 0, 6 and 12 were available for 14 and 11 men, respectively. There was no significant change from baseline in FACT-BP and BPI scores. Change in sCTX was correlated with change in both FACT-BP and BPI scores (r^2^ = 0.64; p  =  0.02 and r^2^ = 0.56; p = 0.02, respectively); change in sCTX was also correlated with change in uNTX (r^2^ = 0.52; p = 0.01).

Eight men experienced nonfatal adverse events during the study period, none of which was judged to be related to ZA. Two men were treated for pneumonia, two had diarrhea, one had an episode of hypotension, one had anemia requiring transfusion, one had deterioration in bone pain and one was transferred to a palliative care unit due to declining performance status. One patient who came off study at 6 weeks due to failure of bone suppression died of his disease a week later.

## Discussion

ZA is often administered every 3 weeks in men with bone metastases from CRPC. Here, we demonstrate that many men achieve suppression of markers of bone turnover for 12 weeks following ZA. High telopeptide at baseline is known to be correlated with higher burden of bone metastasis [6] and men with high baseline telopeptide levels had a lower chance of suppressed telopeptide levels at 12 weeks. Whether such men might derive benefit from more frequent ZA treatments is unknown although a recent study reported in this journal suggested that bone turnover markers are suitable predictors of mortality risk, disease progression and SREs in men with prostate cancer [7].

Although QoL assessed by FACT-BP and BPI did not change significantly during the study period, there was a correlation between the fall in sCTX concentrations and improvement in QoL scores. Given that bone turnover was suppressed in most men, QoL is largely a function of side effects of treatment. Therefore, less frequent administration of ZA is likely to have favorable effects on men's QoL. However, short study duration may have impacted our results on QoL.

ZA is used to delay or prevent SREs in other tumor types that involve bone such as breast cancer and myeloma. Clinical outcomes of 4- and 12-weekly schedules of ZA in women with breast cancer and bone metastases were shown to be similar [8]. Recently, a large study evaluating monthly versus 3-monthly use of ZA in multiple tumor types was reported at a meeting to demonstrate noninferiority for 3-monthly compared with monthly dosing (29.5 vs 28.6% of participants with SREs at 2 years, respectively). Similar questions apply to the optimal frequency of administration of the RANK ligand inhibitor denosumab and a Phase III clinical trial assessing the efficacy of 4 versus 12 weeks of denosumab is underway [9].

Our study has limitations. It did not have a control arm and the sample size was small; it was conducted at a single tertiary cancer center potentially introducing selection bias. Also, the optimal level of telopeptide suppression to achieve clinical meaningful effects remain unknown. Despite these limitations, we found that the majority of men with CRPC and bone metastases have suppression of markers of bone turnover for at least 12 weeks following their first injection with ZA, suggesting that a less frequent schedule of administration should be investigated in definitive clinical trials with clinical end points.

## Conclusion & future perspective

Based on the markers of bone turnover suppression, we demonstrate that majority of men require less frequent injections of ZA than is currently approved. Given the recent evidence suggesting similar efficacy with 12-weekly ZA compared with 3 weekly, less frequent injections are likely sufficient in most men: individualizing frequency based on baseline risks could help save inconvenience and cost of frequent treatments. Results of studies looking at appropriate frequency of treatment with other bone targeted agents like denosumab (NCT02051218) [9] are awaited.

**Summary points**

- Zoledronic acid (ZA) delays skeletal-related events in men with prostate cancer and bone metastases. It is currently approved for use every 3 or 4 weeks.
- ZA is associated with adverse effects such as necrosis of the jaw, renal impairment and hypocalcemia.
- Given the long half-life of ZA, we aimed to investigate the proportion of men with suppressed urine and serum telopeptides (uNTX and sCTX, respectively) at 12 weeks after first injection of ZA.
- We found that a majority of men had suppressed levels of uNTX and sCTX at 12 weeks after one injection of ZA; there was no change in quality of life in men during this period.
- Most men need less frequent injections of ZA and further studies need to select men who require less versus more frequent injections.
